# Hemoadsorption in the Management of Septic Shock: A Systematic Review and Meta-Analysis

**DOI:** 10.3390/jcm14072285

**Published:** 2025-03-27

**Authors:** David Steindl, Tim Schroeder, Alexander Krannich, Jens Nee

**Affiliations:** 1 Charité Poison Control Center, Charité—Universitätsmedizin Berlin, 1220 Berlin, Germany; david.steindl@charite.de; 2 Department of Nephrology and Medical Intensive Care, Charité—Universitätsmedizin Berlin, 10117 Berlin, Germany; tim.schroeder@charite.de (T.S.); jens.nee@charite.de (J.N.); 3 BioStats GmbH, 14641 Nauen, Germany

**Keywords:** CytoSorb, septic shock, hemoadsorption

## Abstract

**Background/Objectives**: Septic shock remains a significant clinical challenge with consistently high mortality rates. Recent investigations have focused on the efficacy of CytoSorb^®^ (CytoSorbents Corporation, Monmouth Junction, NJ, USA), an extracorporeal cytokine adsorber, and how it impacts outcomes in sepsis. The current meta-analysis reports on the impact of CytoSorb^®^ on survival, specifically in septic shock patients. **Methods**: We conducted a comprehensive systematic search across the PubMed and COCHRANE databases for studies published up to 10 June 2024. The analysis prioritized randomized controlled trials and observational studies with control groups involving septic shock patients while excluding case reports and case series. Nine studies were finally included in our meta-analysis following the initial screening of 115 articles after excluding duplicates and irrelevant entries. **Results**: The meta-analysis was performed on 744 critically ill patients with septic shock from one RCT and eight observational studies. Of these, 449 patients received treatment with CytoSorb^®^ in addition to standard care. Our data indicate that CytoSorb^®^ use is associated with reduced in-hospital mortality, evidenced by an odds ratio (OR) of 0.64 [0.42; 0.97] and a *p*-value of 0.036. For 28–30-day mortality, the findings were more pronounced with an OR of 0.49 [0.28; 0.83] and a *p*-value of 0.003. The analysis of the longest observed mortality showed a trend for improved survival within the CytoSorb group; however, it did not reach statistical significance. Additionally, there was a significant improvement in hemodynamic stability as a secondary endpoint, as evidenced by notable reductions in vasopressor requirements in the hemoadsorption group. **Conclusions**: The current meta-analysis suggests that the use of CytoSorb^®^ alongside standard of care management may be linked to improved short-term survival in patients with septic shock; however, these findings should be interpreted with caution in light of the heterogeneity and the modest quality of the studies included. Prospective studies are needed to better determine the impact of hemoadsorption on shock reversal and survival in these critically ill patients.

## 1. Introduction

Septic shock, a severe manifestation of sepsis, results from a dysregulated immune response to infection, leading to profound circulatory and cellular/metabolic dysfunction. Despite advancements in medical care, sepsis and septic shock continue to pose major public health challenges, with high morbidity and mortality rates. [1]. Recent estimates indicate that sepsis accounts for nearly 11 million deaths annually, disproportionately affecting low- and middle-income countries (LMICs) due to limited healthcare access, delayed diagnosis, and a high burden of infectious diseases. In contrast, high-income countries (HICs) continue to face sepsis-related challenges driven by aging populations, the increased use of invasive medical procedures, and rising antimicrobial resistance [2]. The underlying mechanisms of septic shock involve a dynamic interaction between pro-inflammatory and anti-inflammatory mechanisms, often culminating in a cytokine storm and subsequent multi-organ dysfunction [3]. The innate immune response triggers both pro- and anti-inflammatory signaling pathways, leading to the release of various cytokines and chemokines [4] alongside the activation of alveolar macrophages, which contributes to endothelial damage and alveolar injury [5]. Despite ongoing research and treatment improvements, effectively modulating the overwhelming immune response continues to be a central issue [6]. From its initial identification to current treatment standards, numerous studies have aimed to define and optimize effective therapeutic approaches.

The hypothesis that hyperinflammation results from a dysregulated host immune response serves as the theoretical foundation for employing extracorporeal cytokine removal as an adjunctive treatment option in refractory septic shock as part of the standard of care [7]. This therapeutic approach has increasingly been used over recent years as an adjunctive treatment for immunomodulation in patients with septic shock, aiming to support the balance of the immune system by eliminating excessive levels of both pro-inflammatory and anti-inflammatory mediators.

CytoSorb^®^ (CytoSorbents Corporation, Monmouth Junction, NJ, USA) is an extracorporeal blood purification technology that has received Conformité Européenne (CE) mark approval and is specifically engineered to capture and remove excess cytokines and other inflammatory mediators with molecular weights of up to 60 kDa from whole blood [8]. This system utilizes adsorbent beads composed of polystyrene divinylbenzene, coated with a biocompatible polyvinylpyrrolidone layer, enabling the effective adsorption of smaller molecules through direct retention via various pore sizes, as well as through mechanisms such as hydrophobic interactions, electrostatic forces, hydrogen bonding, and van der Waals interactions [9]. The ability of CytoSorb^®^ to significantly reduce circulating inflammatory cytokines and eliminate proteins that damage the glycocalyx and endothelium has been demonstrated in vitro [10], confirmed through animal studies [11], and, more recently, validated in vivo [12].

Even though hemadsorption has gained increased clinical adoption in recent years, evidence-based support for its efficacy and precise clinical applications remains inconclusive. In the present meta-analysis, we focused specifically on patients with septic shock and analyzed the currently available literature to evaluate the impact of adjunctive CytoSorb^®^ treatment on mortality and shock reversal.

## 2. Material and Methods

This systematic review was pre-registered with PROSPERO under the identifier CRD42024555885. The study was carried out in accordance with the guidelines outlined in the Cochrane Handbook for Systematic Reviews of Interventions, Version 6.4 [13]. The results were reported in accordance with the Preferred Reporting Items for Systematic Reviews and Meta-Analyses (PRISMA) 2020 Statement [14].

### 2.1. Selection Criteria

Included were randomized controlled trials (RCTs) and observational studies involving both pediatric and adult patients with sepsis of any origin who were treated with CytoSorb^®^ and had a control group for comparison. Case reports and case series were not considered. Eligible studies were required to report mortality outcomes. The intervention group had to receive a minimum of one treatment session with the CytoSorb^®^ adsorber, while the control group did not receive any CytoSorb^®^ treatment. Both the intervention and control groups needed to comprise at least three patients. No restrictions were placed on language.

### 2.2. Primary and Secondary Outcomes

The main outcome assessed in this meta-analysis was mortality across different time points. Secondary outcomes were composed of ICU and hospital length of stay, the duration of mechanical ventilation, vasopressor requirements, lactate levels, and SOFA scores. These were analyzed by evaluating both the changes in values before and after CytoSorb^®^ treatment, as well as the differences between the CytoSorb^®^ and control groups.

### 2.3. Search Methods

A systematic literature search was conducted in PubMed and COCHRANE on 10 June 2024 using the following key terms: “CytoSorb AND septic shock AND control group OR randomized”. Studies were included if they investigated CytoSorb^®^ in adult or pediatric septic shock patients with a control group, encompassing both RCTs and observational studies. Exclusion criteria included studies unrelated to CytoSorb^®^, case reports, case series, animal studies, those without a control arm, or lacking original data. Reference lists of relevant articles were screened for additional studies. Filters were applied to exclude studies focusing on non-septic shock conditions, non-interventional studies, and those without clinical outcome data.

### 2.4. Study Selection and Data Extraction Process

Data extraction followed a structured approach using the COCHRANE handbook for systematic reviews. Data extraction and evaluation were performed independently by two of the co-authors. There were no cases of non-consensus. For the selection criteria, studies were included if they reported on hemadsorption treatment in patients diagnosed with septic shock, utilizing the CytoSorb^®^ adsorber specifically for this indication, and with at least one application per patient. The studies had to feature a control group that received standard therapy without hemadsorption. Studies excluded from the analysis were those that did not focus exclusively on septic shock as an inclusion criterion, as well as those where CytoSorb^®^ was not used specifically for this indication, and those without appropriate comparison groups or only single-group analyses.

### 2.5. Assessment of Bias Risk and Evidence of Certainty in Included Studies

The study design, sample size, and treatment allocation of the individual studies included were briefly analyzed and the assessment was conducted at the study level. The risk of bias in included studies was assessed using established tools. The Cochrane risk-of-bias tool (RoB 2) was used to assess the risk of bias in individual randomized trials included in the analysis. The risk of bias in non-randomized studies of interventions (ROBINS) tool was used to assess the risk of bias in individual observational studies with interventions [15,16,17]. To ensure consistency in the data extraction and quality assessment, two reviewers independently screened the studies, extracted data, and assessed the quality of studies using ROBINS-I for observational studies and RoB2 for RCTs. In cases of disagreement, a structured resolution process was followed: reviewers first discussed discrepancies to reach a consensus. If disagreement persisted, a third senior reviewer was consulted to provide an independent assessment and make a final decision. This approach minimized bias and ensured an objective and reliable evaluation of the included studies.

Selection between the common-effect and random-effects models was guided by the degree of heterogeneity in the included studies. Specifically, when the heterogeneity test yielded a *p*-value < 0.05, indicating significant variability across the studies, a random-effects model was applied to account for between-study differences. Conversely, when no significant heterogeneity was detected (*p* ≥ 0.05), a common-effect model was used, assuming a shared underlying effect size across the studies.

### 2.6. Data Synthesis and Statistical Analysis

Odds ratios were used as effect measures. Both common-effect and random-effect meta-analyses were utilized to combine the results from individual studies to improve the precision of treatment effects. The Mantel–Haenszel method [18] was used to estimate polled estimators. Tests for the heterogeneity of studies were performed to check the homogeneity among individual studies. In the case of a significant heterogeneity test (*p* < 0.05), the random-effect estimator was used. In all other cases, a common-effect estimator was used. A forest plot and a funnel plot were presented to visually examine the pooled results and publication bias. Furthermore, bias regression was used to investigate potential bias. The statistics software R version 4.4.1 (R Core Team (2021), R: A language and environment for statistical computing, and R Foundation for Statistical Computing, Vienna, Austria) was used for the meta-analysis calculation.

## 3. Results

**Figure 1:**
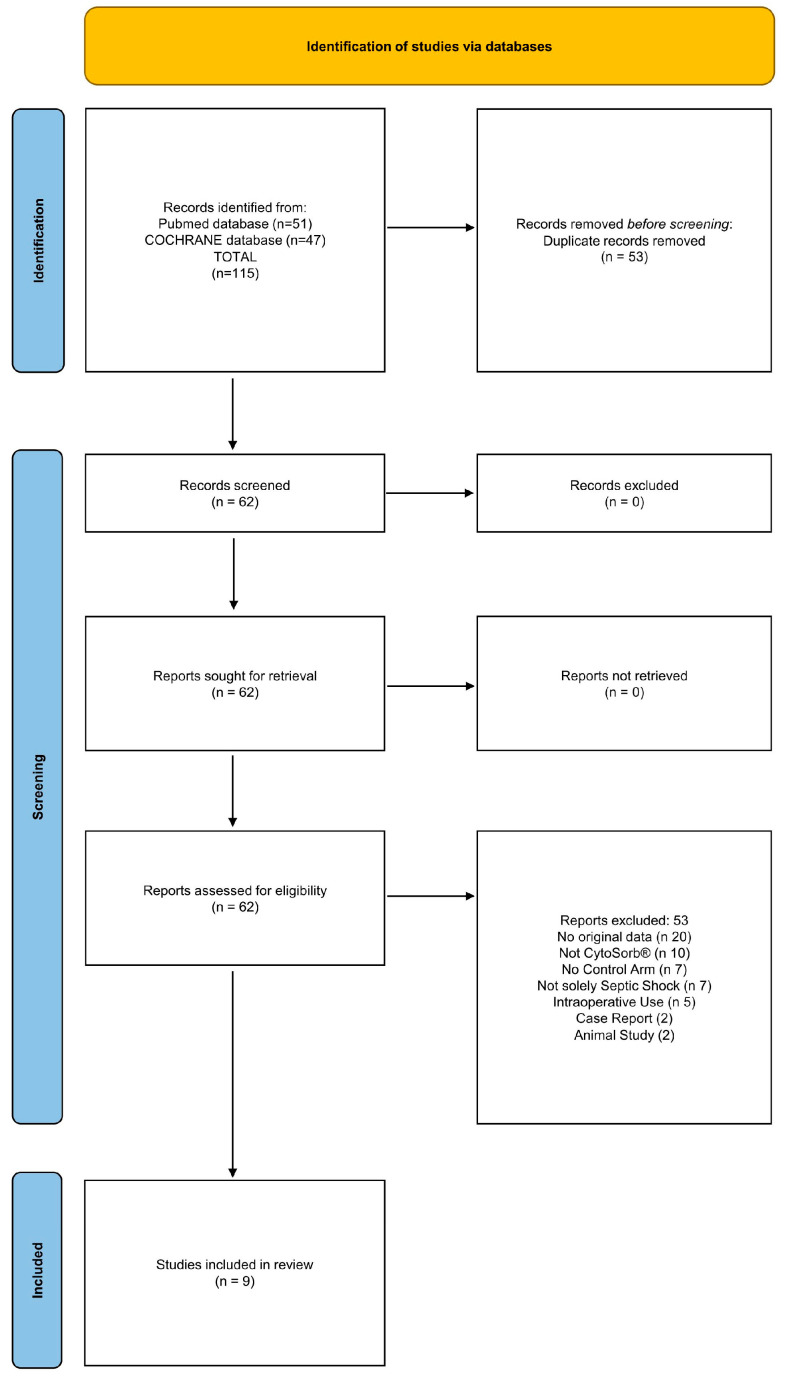
PRISMA 2020 flow diagram: an overview of the search and study selection process [14].

In total, 115 articles were identified (Figure 1). After discarding duplicates (53) and exclusions (53), nine articles remained that fulfilled the inclusion criteria for the meta-analysis, including one RCT and 8 observational studies. Reasons for exclusions were as follows: a lack of original data (20), no use of CytoSorb^®^ (10), no control arm (7), no focus on septic shock, or a focus not solely on septic shock (7), intraoperative use (5), animal studies (2), and case reports (2). Table 1 provides a summary of the included articles, studies, and their key characteristics. The total analytical sample is composed of 744 critically ill patients with septic shock, including 30 pediatric patients (17CS/13CG), with 449 receiving adjunct CytoSorb^®^ with standard-of-care sepsis management, and 295 patients receiving standard-of-care only.

### 3.1. Risk of Bias Assessment and Quality Assessment

The risk of bias assessment and quality assessment of the studies was performed separately for non-randomized studies and randomized trials. The range of bias varied from low to serious. There was no study with a critical risk of bias. The results are shown in Supplementary Figures S12 and S13.

### 3.2. Primary Outcomes

**Figure 2:**
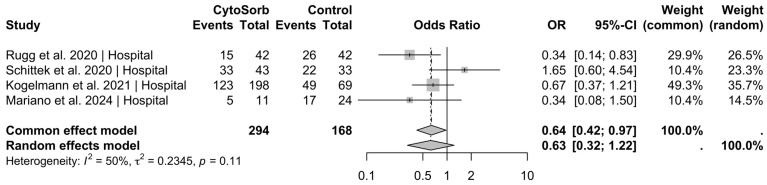
In-hospital mortality. A pooled analysis of data from four studies (total patient count, n = 462) reporting on in-hospital mortality indicated that the use of CytoSorb^®^ was associated with a statistically significant reduction in mortality in the common effect model (OR = 0.64, 95% CI [0.42; 0.97], *p* = 0.036) [22,23,25,28].

**Figure 3:**
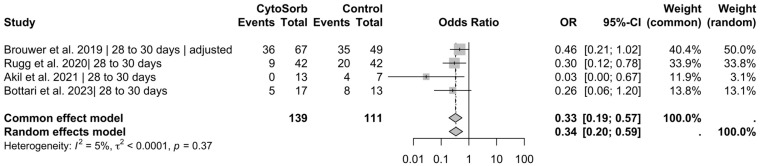
Results for 28 to 30 days of mortality. Pooled data from 4 publications with a total of 250 patients showed that using CytoSorb^®^ was associated with a significant reduction in mortality using the common effect model, with an odds ratio of 0.33 (95% CI [0.19; 0.57], *p* < 0.001 [20,22,24,27].

**Figure 4:**
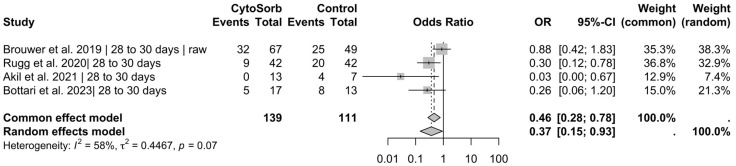
Results for 28 to 30 days of mortality (raw Data). Brouwer et al. also reported mortality data prior to propensity score matching. Therefore, mortality analysis was additionally conducted using these raw data [20,22,24,27].

In the pooled analysis of four studies reporting on in-hospital mortality (n = 462 included patients), CytoSorb® use was significantly associated with reduced mortality under the common-effect model (OR = 0.64, 95% CI [0.42; 0.97], *p* = 0.036) (Figure 2). Pooled data from four studies reporting on 28- to 30-day mortality (n = 250) demonstrated a statistically significant reduction in mortality associated with CytoSorb® treatment under the common-effect model (OR = 0.33, 95% CI [0.19; 0.57], *p* < 0.001) (Figure 3). In this setting, the test for heterogeneity showed no statistical significance (*p* = 0.07); therefore, the common effect model was used, resulting in an odds ratio of 0.46 (95% CI [0.28; 0.78], *p* = 0.003) (Figure 4). However, mortality reduction would have been statistically significant when also using the random effects model, yielding an odds ratio of 0.37 (95% CI [0.15; 0.93], *p* = 0.036).

Moreover, the longest reported mortality follow-up, as well as ICU mortality, were analyzed. Due to the heterogeneity of the included studies regarding the longest reported mortality, the random effects model was utilized, yielding a *p*-value of 0.153. The common effect model narrowly missed statistical significance with a *p*-value of 0.056. Additionally, the analysis of ICU mortality showed no statistical significance in both effect models, with a *p*-value of 0.301 for the common effect model and 0.384 for the random effect model. The respective forest plots are provided in Supplementary Materials.

### 3.3. Secondary Outcomes

Table 2 presents the results of the six studies that reported data on hemodynamic stabilization in patients with septic shock. The changes in norepinephrine dosage, as well as vasopressor requirements and the Vasoactive–Inotropic Score (VIS), were measured at different time points across multiple studies and presented either as within-group or between-group comparisons. The statistical significance levels of the results and the association of CytoSorb^®^ treatment with mortality were also provided. The studies predominantly showed a significant reduction in norepinephrine dosage and vasopressor requirements/VIS after the initiation of CytoSorb^®^ therapy; however, Wendel-Garcia (2021) did not observe a significant change in vasopressor requirements (*p* = 0.555) and also reported no mortality benefit in the intensive care unit (ICU) [26].

### 3.4. Outcomes with Insufficient Reporting

In this review, secondary endpoints such as ICU stay duration, hospital length of stay, lactate levels, SOFA score, and the duration of mechanical ventilation were recorded in at least two studies; however, the paucity of both pre- and post-intervention data, specifically a lack of comparable pre-/post-mean values and standard deviations, precluded their inclusion into the meta-analysis. Despite the absence of a comprehensive quantitative evaluation for these parameters, it should be noted that there was no indication of a negative impact of CytoSorb treatment in the studies that performed these measurements. Rather, a positive trend was observed for ICU as well as hospital length of stay, ventilator-support duration [23], and lactate levels in some of the studies [21,24], suggesting that while these findings were not amenable to meta-analytic review, they might still indicate beneficial outcomes worth noting in clinical contexts.

## 4. Discussion

The present meta-analysis aimed to examine clinical outcomes with the use of hemadsorption as adjunctive therapy as a standard of care treatment in patients with septic shock. Therefore, studies were excluded if they did not specifically focus on septic shock. Despite being the largest RCT on CytoSorb in critically ill patients to date, the study by Schädler et al. [29] was not included in our analysis. The target patient population in this trial consisted of ARDS patients, without septic shock being an inclusion criterion, with a lack of focus on septic shock also evidenced by the fact that the study did not report on any hemodynamic parameters, lactate levels, or data on cardiovascular support medications.

There were two main observations from this meta-analysis. First, there was a significant association between CytoSorb^®^ use and improved short-term survival. Second, we found a significant association between CytoSorb^®^ use and improved hemodynamic stabilization as a marker of shock reversal.

Over the past decade, hemoadsorption has gained growing attention as an adjunctive therapy for various critical care conditions linked to hyperinflammation, with a particular focus on the efficacy of CytoSorb^®^ in managing septic shock, which continues to be a topic of active research and debate. [8]. However, to this date, only a few RCTs and observational studies on hemoadsorption in septic shock have been performed, and high-quality data are still lacking. While several studies have demonstrated positive outcomes regarding cytokine reduction and clinical improvements, the impact on mortality rates in heterogenous patient populations still remains inconclusive [30].

While CytoSorb^®^ has gained increasing attention as a hemoadsorption therapy for septic shock, other extracorporeal blood purification techniques, such as high-volume hemofiltration (HVHF) and polymyxin B hemoperfusion (PMX-HP), have also been explored for their immunomodulatory effects. HVHF aims to reduce inflammatory mediators by increasing convective clearance and potentially improving hemodynamic stability, yet clinical evidence remains inconsistent regarding its survival benefits in septic shock patients. Similarly, PMX-HP selectively binds endotoxins and has demonstrated potential in endotoxin-driven sepsis, particularly in Gram-negative bacterial infections; however, conflicting trial results have raised questions about its overall efficacy. Compared to these methods, CytoSorb^®^ offers a broader spectrum of cytokine removal, independent of endotoxin presence, and does not require high-volume fluid exchange, potentially making it more versatile in various septic shock scenarios. Nonetheless, direct comparative studies between CytoSorb^®^ and other extracorporeal therapies are scarce.

The present systematic review and meta-analysis detected a significant signal towards improved hospital and 28–30-day mortality and reduced vasopressor requirements after HA application. Interestingly, all studies describing a survival benefit for CytoSorb^®^ patients also detected a significant hemodynamic improvement, either within the CytoSorb^®^ group, compared to the control group, or both. This suggests that hemodynamic stabilization and shock reversal achieved through CytoSorb^®^ treatment may potentially be linked to improved mortality outcomes. The exact underlying mechanisms that may explain the favorable survival outcomes identified by our meta-analysis are not entirely clear and are beyond the scope of our investigation. However, the observed mortality benefit in CytoSorb^®^-treated patients may be explained by several physiological mechanisms. One key factor appears to be hemodynamic stabilization, as reflected by reduced vasopressor requirements, which is a commonly reported finding across the included studies. Excessive inflammation and cytokine-induced vasodilation contribute to refractory hypotension and impaired tissue perfusion in septic shock. By removing excess pro-inflammatory mediators, CytoSorb^®^ may help restore vascular tone, leading to improved circulatory stability and a reduced need for vasopressors. Next to its positive effects on macro-hemodynamic stability and shock reversal identified by us and others before us [21], several previous investigations have also demonstrated the potential beneficial effects of CytoSorb hemoadsorption on endothelial integrity [31,32] and microcirculation [33], which, together with improved macro-hemodynamic stability, may contribute to the faster restoration of tissue perfusion and organ function, ultimately promoting improved outcomes.

Furthermore, persistent hyperinflammation is known to drive endothelial dysfunction and multi-organ failure. By modulating the cytokine storm, CytoSorb^®^ may reduce secondary organ damage, thereby improving organ function recovery and overall short-term survival. However, the precise pathways remain incompletely understood, and further studies are needed to determine whether these physiological improvements translate into long-term mortality benefits and organ recovery.

### 4.1. Previous Findings

In 2023, Becker et al. [30] published a meta-analysis and systematic review assessing the efficacy of CytoSorb^®^ across various indications, including sepsis, cardiopulmonary bypass (CPB) surgery, severe illnesses, SARS-CoV-2 infection, and post-cardiac arrest recovery. Their primary endpoint was the longest reported mortality follow-up, with the secondary endpoints comprising ICU length of stay, vasopressor requirements, as well as lactate and IL-6 levels. All endpoints were analyzed both according to the indication for CytoSorb^®^ use and type of study (RCT and observational studies with and without propensity score matching). The main conclusion of the authors was that the available evidence did not support a mortality reduction with CytoSorb^®^ treatment across all evaluated conditions. Unlike the present meta-analysis, Becker et al. did not limit their analysis in the “sepsis” group to patients undergoing septic shock only, which represents a significant difference in this analysis. They highlighted the need for well-designed RCTs targeting specific medical conditions and patient populations, emphasizing the importance of identifying the patients most likely to respond to therapy, such as those with very high cytokine levels and determining the optimal timing for therapy. In the context of evaluating the impact of CytoSorb^®^ on mortality in septic shock, Schmidt et al. [34] explained in their response to Pappalardo et al. [35] the significance of mortality as a primary endpoint in meta-analyses, due to its objective and quantifiable properties. The authors argued that individual studies may not always directly demonstrate a mortality benefit, but the collective evidence from meta-analyses can reveal significant effects [34]. This perspective is central to our meta-analysis as it underscores the need for further research to substantiate the use of CytoSorb^®^ and its potential benefits in reducing mortality among patients with septic shock.

### 4.2. Current Analysis

Unlike the Becker et al. meta-analysis, which included a heterogeneous array of studies [30], our analysis specifically focused on septic shock, which may explain the difference in the results compared to our findings. Our analysis of nine studies indicates significant evidence supporting the efficacy of CytoSorb^®^ in reducing both hospital and 28–30-day mortality, specifically in septic shock patients. Hospital mortality was reduced by 36%, and 28–30-day mortality saw a reduction of 51% in the raw analysis and even 66% in the adjusted analysis. These results underscore the potential of CytoSorb^®^ to enhance short-term survival in septic shock patients. We, therefore, believe that appropriate patient selection is a major key component for the successful application of adjunctive CytoSorb^®^ treatment and that the same may exhibit its most reproducible benefit in critically ill and severely unstable patients rather than in heterogenous, unselected “all comer” populations [8]. However, our conclusions must be interpreted cautiously due to the limited number and quality of the studies analyzed. Our risk of bias assessment using ROBINS-I for observational studies and RoB2 for randomized controlled trials identified varying degrees of bias among the included studies (Supplementary Figures S12 and S13). While the overall trend suggests a potential survival benefit, the presence of bias, particularly in observational studies, necessitates cautious interpretation. Sensitivity analyses were conducted to assess the robustness of our findings, but further high-quality, well-controlled RCTs are needed to confirm the observed effects.

### 4.3. Safety

The included studies did not report any major treatment-related adverse events. Thrombocytopenia, a potential side effect associated with all extracorporeal therapies, was identified as a possible concern [36]. This was reported in one of the studies [22]; however, it remained unclear whether this effect was attributable to hemoadsorption itself or if it was a consequence of extracorporeal circulation. Importantly, no resulting clinical consequences or bleeding events were reported in association with thrombocytopenia, which is in line with the results from other research groups [37].

Since CytoSorb^®^ eliminates not only cytokines but also a range of other substances, there is a theoretical concern regarding the unintended removal of essential molecules, such as specific medications. This concern, which applies to all extracorporeal therapies, is influenced by drug-specific factors, including the volume of distribution, protein binding, and elimination of the half-life. In drugs prone to adsorption, CytoSorb^®^ adsorption kinetics indicate that the highest adsorption occurs within the initial hours, suggesting a potential need for an increased loading dose and/or supplementary dosing of certain medications within the first 1–2 h of treatment. [32]. However, data provided by Schneider et al. demonstrate the limited impact of CytoSorb^®^ on the pharmacokinetics of most anti-infective drugs tested, as also reported for meropenem [38] and amikacin [39]. The clearance of some drugs like fluconazole, amphotericin B, linezolid, and vancomycin may be increased [29,40]. While providing specific dosing recommendations is beyond the scope of this meta-analysis, there is a growing body of in vivo research investigating the pharmacokinetics of commonly used anti-infective agents under CytoSorb^®^ therapy, offering increasingly refined guidance for clinical decision-making [38,40,41]. Future studies should continue to explore evidence-based dosing strategies to optimize antimicrobial therapy in patients receiving CytoSorb^®^

### 4.4. Strengths and Limitations

As far as we are aware, this represents the first systematic review and meta-analysis focusing on hemadsorption with CytoSorb^®^ specifically in septic shock patients. While our results are encouraging, there are a number of limitations that need to be acknowledged that limit the validity and the generalizability of our results. Our meta-analysis is significantly limited by the small number, heterogeneity, and limited quality of available studies. The lack of randomized trials, or trials with minimized confounding factors, further weakens the robustness of our findings, resulting in a low level of evidence. Additionally, the limited number and quality of studies restricted our ability to conduct sufficiently powered pooled analyses on various outcomes, and we cannot exclude the possibility of publication bias. By the nature of their design, meta-analyses and systematic reviews contain studies with a variety of different patient selection criteria and treatment modalities, and also a certain variability in the reported outcome measures in the included publications. Furthermore, as with any analysis that includes retrospective observational data, there is an inherent bias that cannot be completely accounted for. Of note, none of the trials included in our analysis, including the RCT, blinded the clinical personnel. Due to the nature of the intervention, blinding is difficult to establish for extracorporeal blood purification procedures, and this lack could lead to bias within the treatment of healthcare professional teams, which could exhibit a certain impact on patient management. Another major limitation of our present work is that while all patients underwent septic shock, the treatment modalities of CytoSorb^®^ were largely heterogenous in terms of the point in time when CytoSorb^®^ treatment started in relation to onset of septic shock, the duration of CytoSorb^®^ treatment, and adsorber exchange intervals. Furthermore, except for the presence of septic shock, the exact patient selection criteria for CytoSorb, e.g., in terms of vasopressor dose, the lactate level, and extent of hyperinflammation, have been heterogenous and, in part, not reported in detail. Another notable limitation is our inability to assess safety outcomes in detail, such as bleeding, platelet count, and drug removal. It is important to note, however, that regarding these safety concerns, nothing relevantly negative was reported in the studies included in our analysis. Moreover, the absence of sufficiently reported data on relevant clinical outcomes, such as durations of hospital stay and mechanical ventilation, necessitates a cautious interpretation of mortality-related findings. From a statistical perspective, it is essential to acknowledge that while estimating the mean and standard deviation from quartiles is a widely accepted method in meta-analyses, these calculations remain approximations of the actual values.

### 4.5. Implications of This Meta-Analysis and Future Directions

Despite the above-mentioned limitations, our meta-analysis found decreased short-term mortality in critically ill patients with septic shock receiving CytoSorb. In the absence of well-designed, prospective RCTs, the clinical significance of these results should be interpreted with caution but should also not be ignored. The fact that, unlike a recently published meta-analysis in heterogenous patient populations [30], our analysis focusing on septic shock indicated a survival benefit for CytoSorb^®^ patients underscores the importance of appropriate patient selection for adjuvant CytoSorb^®^ treatment, rather than its broad-scale use in unselected patient groups.

Further research, particularly large-scale, multicenter randomized controlled trials, is essential to confirm the observed benefits and to better understand the optimal patient selection and treatment protocols for CytoSorb^®^ in septic shock.

Given its potential to stabilize hemodynamics and reduce vasopressor requirements, CytoSorb^®^ may serve as a valuable adjunct to existing sepsis treatment protocols. While standard management remains centered on early antimicrobial therapy, fluid resuscitation, and vasopressor support, extracorporeal cytokine removal could offer additional therapeutic benefits, particularly in patients with refractory septic shock. To address this, standardized criteria for optimum timing, dosage, and duration of hemoadsorption treatment should be more thoroughly explored.

Future studies should also aim to address long-term outcomes and potential safety concerns, including the impact on drug pharmacokinetics and other adverse events. Understanding the optimal timing and duration of therapy, along with potential interactions with other treatments, will be vital for the further elucidation of the therapeutic effects and outcomes of CytoSorb^®^ hemoadsorption [8].

Lastly, future clinical protocols may benefit from stratifying patients based on inflammatory biomarkers, hemodynamic parameters, and severity scores (e.g., SOFA or APACHE II). By identifying those most likely to benefit from CytoSorb^®^ therapy, clinicians can optimize outcomes while minimizing unnecessary interventions.

## 5. Conclusions

The findings of this meta-analysis suggest that CytoSorb^®^ therapy may be associated with improved short-term survival, potentially through hemodynamic stabilization and shock reversal in patients with septic shock. However, the limitations of this study’s quality and heterogeneity warrant a cautious interpretation of these results. Further high-quality, well-controlled studies are essential to conclusively establish the efficacy and safety of hemoadsorption in sepsis management.

## Figures and Tables

**Table 1:** Baseline characteristics of the included articles.

	Author	Year	Study Design	N (CS)	N (CG)	Median Age (CS/CG)	Sex; Male (%) CS/CG
1	Brouwer et al. [19,20]	2019/2021	Retrospective IPTW-weighted study; single-center study	67	49	61/69	55/61
2	Hawchar et al. [21]	2019	Randomized controlled trial (RCT), open-label, single-center study	10	10	60/71	70/60
3	Rugg et al. [22]	2020	Retrospective propensity score-matched study; single-center study	42	42	64/68	64/60
4	Schittek et al. [23]	2020	Retrospective control group with a prospective intervention group; single-center study	43	33	63/62	88/72
5	Akil et al. [24]	2021	Single-center study with a retrospective control group and a prospective intervention group	13	7	61/61	38/29
6	Kogelmann et al. [25]	2021	Retrospective study; multicenter study	198	69	62/66	61/NA
7	Wendel-Garcia et al. [26]	2021	Single-center propensity score-matched study with a retrospective control group and a prospective intervention group	48	48	57/58	65/65
8	Bottari et al. [27]	2023	Retrospective control group with a prospective intervention group; single-center study	17	13	9/6	8/11
9	Mariano et al. [28]	2024	Retrospective study; single-center study	11	24	63/72	8/20
	Total:			449	295		

Legend: CS: CytoSorb^®^; CG: control group; IPTWs: inverse probability of treatment weights; RCT: randomized controlled trial, NA: not available.

**Table 2:** Vasopressor requirements.

Study	Change in Vasopressor Dosage or VIS in CytoSorb^®^ Group	Time Window of Measurement	Statistical Significance Within CytoSorb^®^ Group	Statistical Significance Between Groups	Mortality Benefit CS
Akil et al. (2021) [24]	Significant reduction at 12, 24, and 48 h; none after 72 h	12, 24, 48, 72 h	*p* < 0.001	<0.03 ^#^	Yes, 30 days
Rugg et al. (2020) [22]	Halving of median NE equivalents to 0.26 μg/kg/min	24 h	NR	<0.01 ^#^	Yes, hospital and 28 days
Hawchar et al. (2019) [21]	Reduction from 0.54 to 0.16 μg/kg/min	48 h	*p* = 0.016	<0.35 ^#^	Yes, 48 h
Wendel-Garcia et al. (2021) [26]	NR, reduction in requirement	72 h	NR	*p* = 0.555	No
Bottari et al. (2023) [27]	Significant reduction in VIS	72 h	NR	*p* = 0.001	Yes, 28 days
Mariano et al. (2024) [28]	Significant reduction at various time points	Days 1–4	NR	*p* = 0.02	Yes, hospital

^#^ *p*-value estimated. Legend: NE: norepinephrine; VIS: Vasoactive–Inotropic Score; CS: CytoSorb; NR: not reported.

## Data Availability

Not applicable.

## Supplementary Materials

The following supporting information can be downloaded at: https://www.mdpi.com/article/10.3390/jcm14072285/s1, Figure S1. Funnel plot Endpoint hospital mortality; Figure S2. Funnel plot Endpoint 28 to 30 days mortality (Brouwer adjusted); Figure S3. Funnel plot Endpoint 28 to 30 days mortality (Brouwer raw); Figure S4. forest plot Endpoint ICU mortality; Figure S5. Funnel plot Endpoint ICU mortality; Figure S6. Forest plot Longest mortality (Brouwer adjusted); Figure S7. Funnel plot Longest mortality (Brouwer adjusted); Figure S8. Forest plot Longest mortality (Brouwer raw); Figure S9. Funnel plot Longest mortality (Brouwer raw); Figure S10. Forest plot Longest mortality inc. 48 h and 1 year; Figure S11. Funnel plot Longest mortality inc. 48 h and 1 year; Figure S12: Risk of Bias in Non-randomized Studies of Interventions; Figure S13: Risk of Bias in randomized trials; Table S1. Endpoint assessment referring partial on GRADE.
