# Supplementary material for: Hemoadsorption in the Management of Septic Shock: A Systematic Review and Meta-Analysis
Source: J Clin Med. 2025 Mar 27;14(7):2285. doi: 10.3390/jcm14072285 (PMC11989519; doi:10.3390/jcm14072285)
Supplement: Supplementary file 1 [file jcm-14-02285-s001.zip › jcm-3505942-supplementary.pdf]

Supplement Figure S1. Funnel plot Endpoint hospital mortality

Linear regression test of funnel plot symmetry, p = 0.88

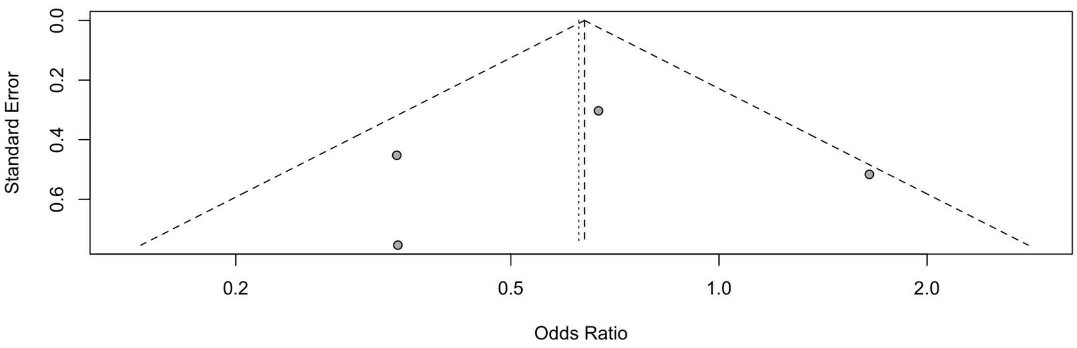

Supplement Figure S2. Funnel plot Endpoint 28 to 30 days mortality (Brouwer adjusted)

Linear regression test of funnel plot symmetry, p = 0.053

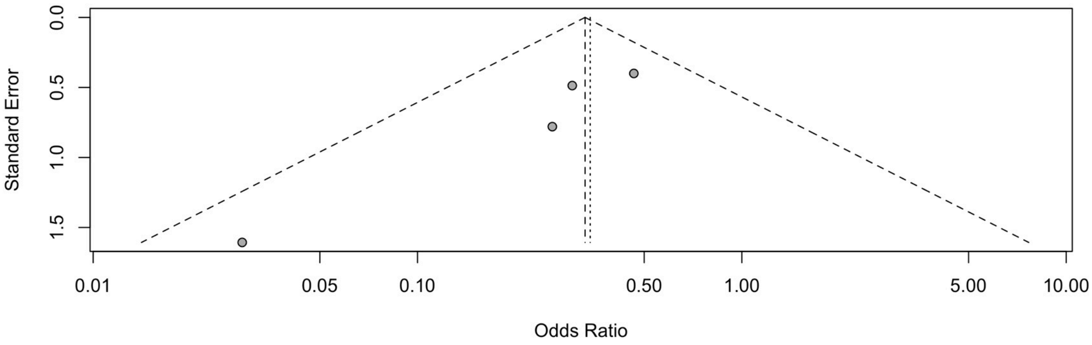

Supplement Figure S3. Funnel plot Endpoint 28 to 30 days mortality (Brouwer raw)

Linear regression test of funnel plot symmetry, p = 0.121

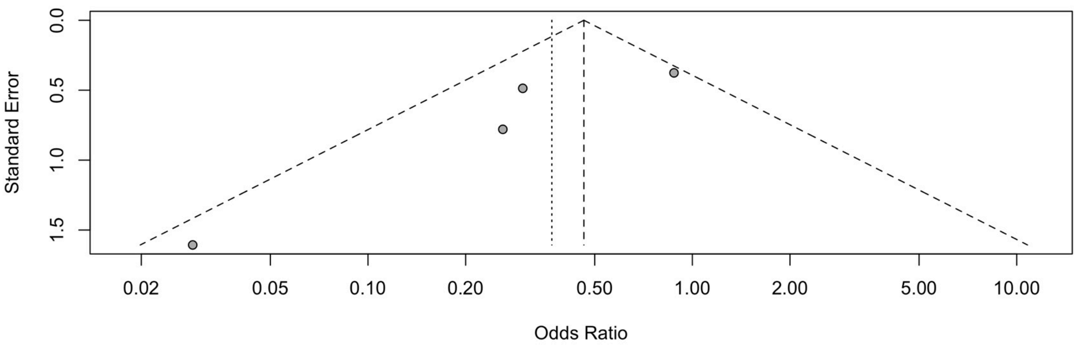

Supplement Figure S4. forest plot Endpoint ICU mortality

Study	CytoSorb Events	CytoSorb Total	Control Events	Control Total	OR	95%-CI	Weight (common)	Weight (random)
Schittek et al. 2020 | ICU	31	43	22	33	1.29	[0.48; 3.45]	17.4%	27.4%
Kogelmann et al. 2021 | ICU	118	198	44	69	0.84	[0.48; 1.48]	65.9%	40.7%
Wendel-Garcia et al. 2021 | ICU	32	48	20	48	2.80	[1.22; 6.42]	16.7%	31.9%
**Common effect model**		**289**		**150**	**1.24**	**[0.82; 1.88]**	**100.0%**	**.**
**Random effects model**					**1.39**	**[0.66; 2.89]**	**.**	**100.0%**

Heterogeneity: $I^2 = 64\%$, $\tau^2 = 0.2623$, $p = 0.06$

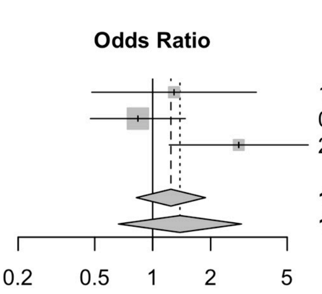

p-value for common effect model: 0.301

Supplement Figure S5. Funnel plot Endpoint ICU mortality

Linear regression test of funnel plot symmetry, p = 0.525

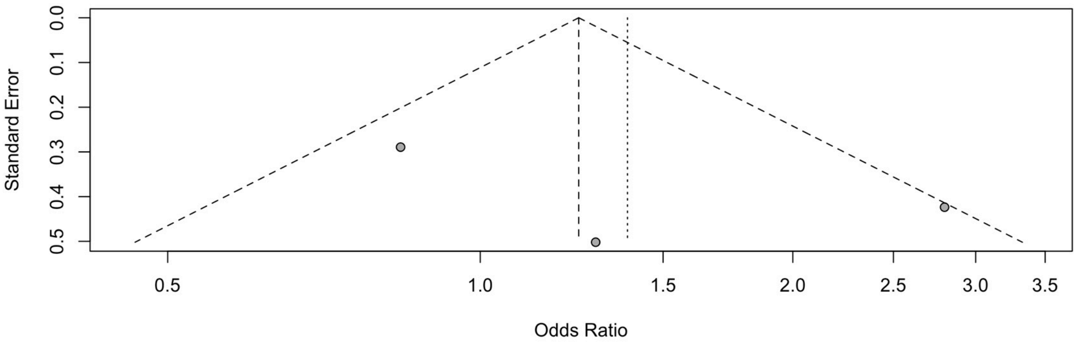

Supplement Figure S6. Forest plot Longest mortality (Brouwer adjusted)

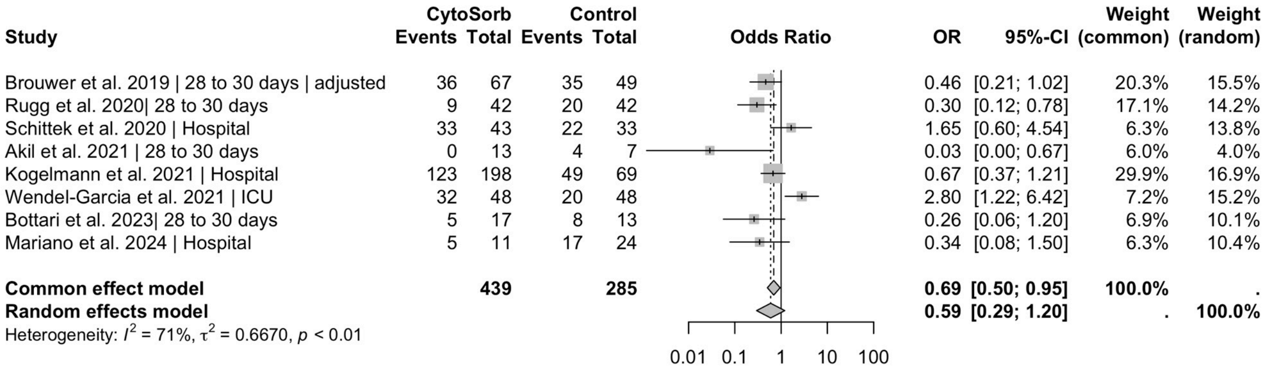

p-value for random effect model: 0.147

Supplement Figure S7. Funnel plot Longest mortality (Brouwer adjusted)

Linear regression test of funnel plot symmetry, p = 0.282

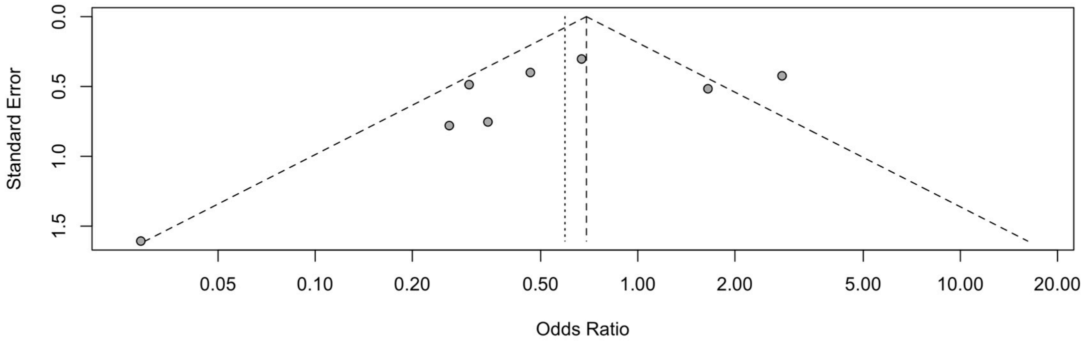

Supplement Figure S8. Forest plot Longest mortality (Brouwer raw)

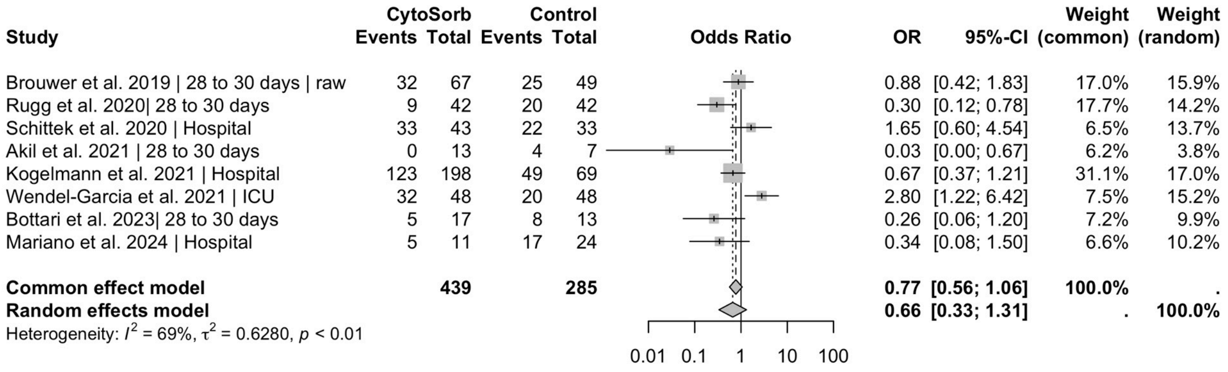

p-value for random effect model: 0.238

Supplement Figure S9. Funnel plot Longest mortality (Brouwer raw)

Linear regression test of funnel plot symmetry, p = 0.227

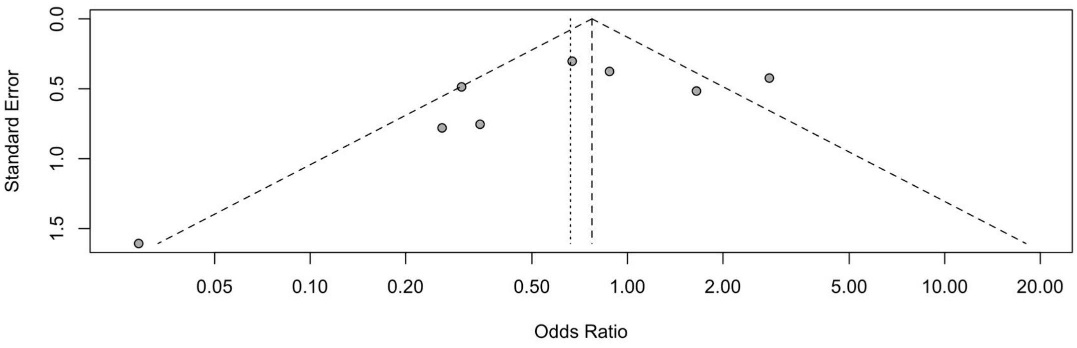

Supplement Figure S10. Forest plot Longest mortality inc. 48 hrs and 1 year

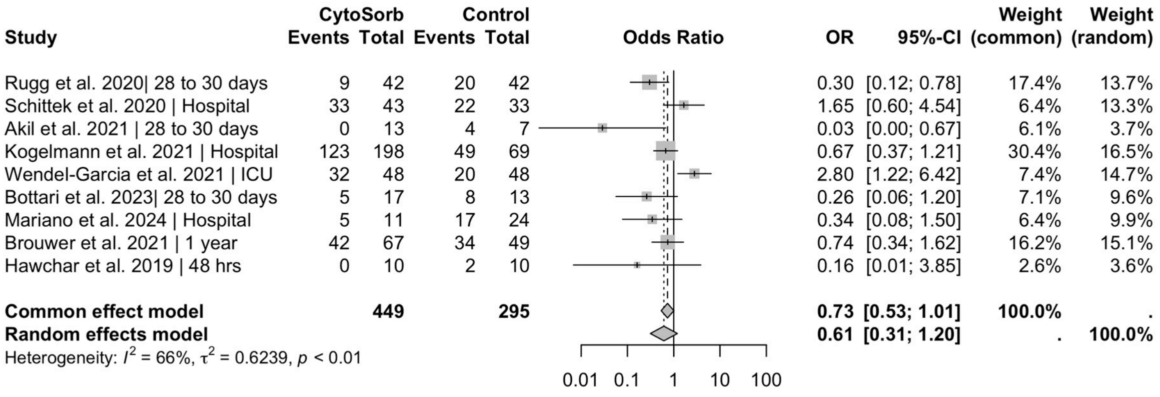

p-value for random effect model: 0.153

Supplement Figure S11. Funnel plot Longest mortality inc. 48 hrs and 1 year

Linear regression test of funnel plot symmetry, $p = 0.176$

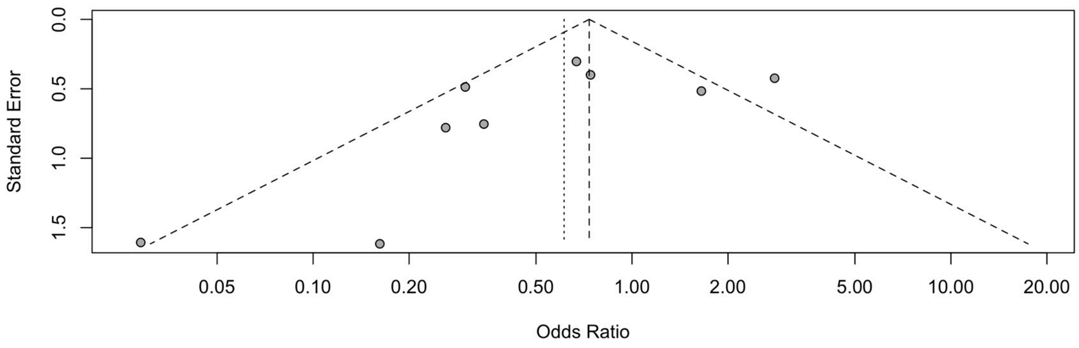

Supplement Figure S12: Risk of Bias in Non-randomized Studies of Interventions

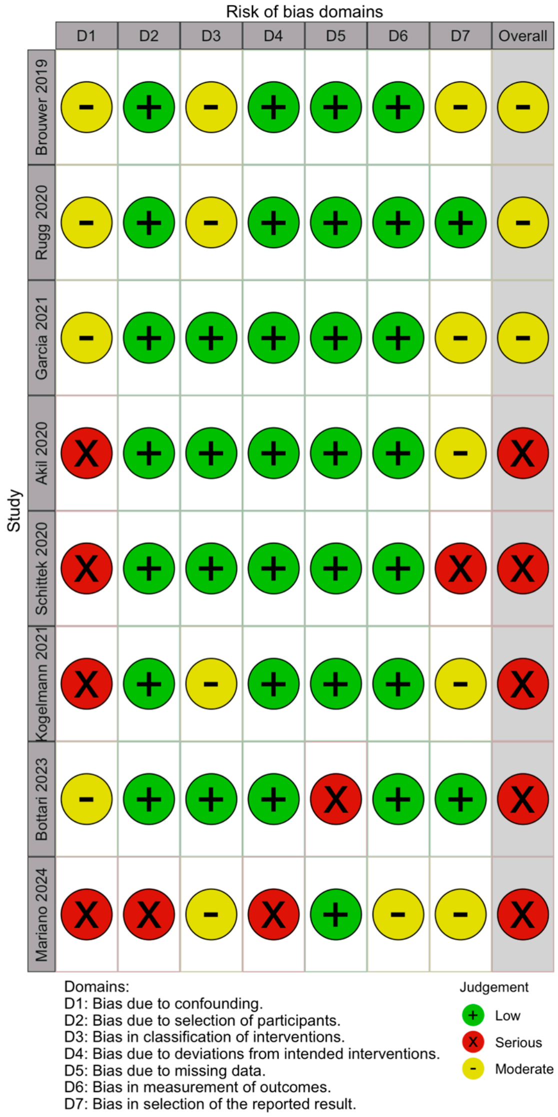

Supplement Figure S13: Risk of Bias in randomized trials

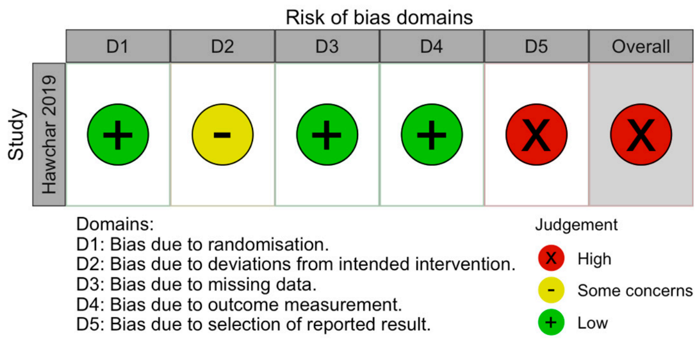

Table S1. Endpoint assessment referring partial on GRADE.

Outcome	Study_Design	Risk of Bias	Inconsistency	Indirectness	Imprecision	Publication Bias	Overall Quality
Mortality	Randomized Controlled Trials (RCTs) / Observational studies	Low	Low	Low	Low	Low	High
